# Biostimulants for Sustainable Management of Sport Turfgrass

**DOI:** 10.3390/plants12030539

**Published:** 2023-01-24

**Authors:** Sara Bosi, Lorenzo Negri, Mattia Accorsi, Loredana Baffoni, Francesca Gaggia, Diana Di Gioia, Giovanni Dinelli, Ilaria Marotti

**Affiliations:** 1 Department of Agricultural and Food Sciences, Alma Mater Studiorum—University of Bologna, 40127 Bologna, Italy; 2 Independent Researcher, 40060 Dozza, Italy

**Keywords:** *Agrostis stoloniferous* L., turf management, biostimulants, microbial inoculum, humic acid, mycorrhizal fungi, thatch control, evapotranspiration, visual quality

## Abstract

Research on the efficacy of innovative, ecofriendly biostimulants in sport turf management is scarce, with less information available from open-field experiments, and even less pertaining to thatch control-related problems. The objective was to investigate the open-field effectiveness of a commercial product, EM-1, and two newly developed products, ExpA and ExpB, in improving both rhizosphere and turfgrass, *Agrostis stoloniferous* L., characteristics on a golf green. ExpA and ExpB, identical in microbial composition, were equally effective in significantly increasing chlorophyll synthesis and visual turf quality, as well as in resistance to tearing out, compared to the untreated control 56 days after treatment (DAT). EM-1 showed intermediate trends between the control and novel biostimulants. The inclusion of humic acids and mycorrhizal fungi to the microbial composition in ExpB significantly improved some rhizosphere properties 56 DAT relative to the control. Results on ExpB evidenced a significant decrease in the thatch layer thickness and fresh leaf weight, associated with a significant increase in the humus thickness, organic matter decomposition and evapotranspiration efficiency. An increased dry leaf biomass was also shown. ExpA and EM-1 showed either marginal or intermediate improvements relative to the control. ExpB represents a promising alternative to alleviate negative environmental impacts associated with turf maintenance-related activities.

## 1. Introduction

The maintenance of high-quality turf (grass cover for football fields, golf courses, home lawns, parks and roadsides) is important in providing positive environmental, social and economic benefits. However, the management of high-quality turf relies on the use of synthetic agrochemicals to meet the high standards for aesthetics and playability, which can have a concomitant strong negative impact on the environment [1,2,3,4]. As with the agricultural sector, negative environmental impacts are currently being addressed through innovative technology, involving the use of environmentally friendly, novel plant biostimulants based on natural substances, including amino acids, plant extracts, humic substances, protein hydrolysates, mycorrhizal fungi and N-fixing bacteria to support plant growth and development [1,4,5,6,7,8,9,10]. Given that European legislation is focusing on the reduction in pesticides and fertilizers by 2030, innovation policies are gaining importance in new technologies, including those related to the manufacture of biostimulants [4].

Despite a rigorous ongoing debate over the last decade to provide a concise and biologically meaningful definition of biostimulants [5,7,8,10], a definition was subsequently provided under the new European Regulation (EU) 2019/1009 as follows: “A plant biostimulant shall be an EU fertilising product the function of which is to stimulate plant nutrition processes independently of the product’s nutrient content with the sole aim of improving one or more of the following characteristics of the plant or the plant rhizosphere: (i) nutrient use efficiency, (ii) tolerance to abiotic stress, (iii) quality traits, or (iv) availability of confined nutrients in the soil or rhizosphere” [11]. From a comprehensive review of the literature [8] on biostimulants for agriculture, it was reported that although studies are scarce, the additive and/or synergistic effects of various biostimulant categories showed that combinations of nonmicrobial biostimulants or microbial inoculants with humic acids, plant extracts or protein hydrolysates provided more reproducible benefits to plant growth and production. In recent years, the use of biostimulants in the field of agriculture has increased [8,10,12], and examples of various commercialised microbial products and patents can be reviewed in Kumar et al. [12]. These products have been tested on cereal crops, sugarcane, sunflowers and rapeseed, as well as on crops that fall within the horticultural subdivision of agriculture, such as vegetables (lettuce and potatoes), fruit (pineapple) and ornamental plants [8,12].

As turfgrass is considered a horticultural crop, many of the biostimulants used in horticulture are being incorporated into turfgrass management programs—although, often, with little understanding [13]. This is because turf management faces unique challenges as the rootzones used for most new sport turf construction are designed with minimal organic matter and are almost completely devoid of microbial life, supporting grass growth in the natural environment [2,6]. Hence, biostimulant-based improvements to the plant rhizosphere, in accordance with regulation (EU) 2019/1009 [11], would necessitate the increased degradation of organic matter and improved humification. In recent years, more attention has been focussed on controlling thatch in turfgrass [14,15,16,17,18]. Creeping bentgrass (*Agrostis stoloniferous* L.), the most widely used cool season turfgrass on golf greens, is a potent producer of thatch, composed of intermingled living and dead plant tissue [19] formed from excess organic matter build-up (predominantly in the form of stolon development) and slow decomposition rates. Excessive thatch is commonly associated with decreased plant rooting, hydraulic conductivity, water filtration and tolerance to cold temperatures, with increased dry zones and disease-related problems [2,19]. This necessitates frequent maintenance activities, which incur significant costs (mowing, topdressing and pesticide application), coinciding with negative environmental impacts [3,17,19]. In turfgrass management, the need for biostimulants for thatch degradation (increase humus production, ameliorate the rhizosphere nutrient status, enhance disease prevention and the maintenance of soil friability) has been considered more essential than the action of the biostimulant on the plant itself [6]. Nonetheless, additional challenges to turf management include the high standards of aesthetics [20].

Despite the many reputed benefits, reliable scientific knowledge of the effects of biostimulants on grasses is considered scarce [4]. The requisite for more research on the effect of biological products on grasses has been highlighted [4,13]. Aside from the European legislation objective in reducing the use of pesticides and fertilizers by 2030, as well as the promotion of new technologies [4], an additional driving force behind the increasing interest in biostimulants for all plants is that of climate change [21]. The use of biostimulants in mitigating the adverse impacts of harsh environmental conditions on plants are currently being reported [21]. As such, the use of biostimulants is projected to increase on a global level by 10.4% annually until 2030 [22]. The application of commercial biostimulants to turfgrass species has been shown to significantly increase in the values of visual parameters, such as the colour (chlorophyll content), as well as in promoting plant development (plant biomass) and in reducing microbial infestation [9,13,23,24]. The efficacy of a commercial product containing effective microorganisms (EMs) in promoting root extension and increased resistance to tearing out was demonstrated for *Lolium perenne* L. [1]. EMs are consortia of beneficial naturally occurring organisms (photosynthesizing bacteria, lactic acid bacteria, yeasts, actinomycetes and fermenting fungi) that can be applied as inoculants to increase the microbial diversity of the soil ecosystem, thereby improving soil ecology and creating an environment favourable to the growth and health of plants [25]. Moreover, commercial humic acid-based biostimulants have, similarly, been shown to positively impact root length, surface area, root volume, biomass and viability on both perennial ryegrass and creeping bentgrass [26,27]. Regarding thatch control and associated problems, there is very little published evidence pertaining to the effectiveness of biostimulants, and more work is required in this area [15,16,17,18].

Given the increased importance in innovative new technologies [4] and the requisite for more open-field investigations to confirm the efficacy of biostimulants in the environmental management of sport fields [1], especially with regard to thatch-associated problems [17,18], the present study aimed at addressing these aspects. Using both a commercial EM-1 product, as well as two additional innovations, the present study evaluated the efficacy of using *Agrostis stoloniferous* L. in the open-field on a golf green. Specifically, improvements to thatch-related problems (thatch humus profile, organic matter, element composition and evapotranspiration) were investigated, along with plant-related improvements (tearing out, leaf biomass, visual quality and photosynthetic pigments).

## 2. Results

### 2.1. Biostimulant Effects on the Plant

The effect of the biostimulant treatments was examined to ascertain any changes to leaf biomass. This comparison was determined for the untreated control, the commercial biostimulant product EM-1, as well as for the two products under investigation. The two experimental treatments were formulated by the Department of Agricultural and Food Sciences—University of Bologna—and labelled as ExpA and ExpB, respectively.

The fresh leaf weight increased from 0 DAT to 28 DAT, and was maintained at 56 DAT with no significant differences between the control and ExpA- and B-treated plots, respectively (Table 1). Fresh mass weight was significantly lower after treatment with EM-1 compared to the remaining treatments. The dry weight increased significantly from 0 DAT to 28 DAT and was maintained at 56 DAT. The dry leaf weight at 56 DAT treated with ExpB was significantly higher than the control and EM1 treatment (Table 1).

Considering that it was reported that various stressful environments reduce the photosynthetic pigment content, these parameters were examined to highlight possible stress conditions in putting greens [28]. Results of the chlorophyll and carotenoid content extracted from *Agrostis stoloniferous* L. leaves are shown in Table 2.

**Figure 1:**
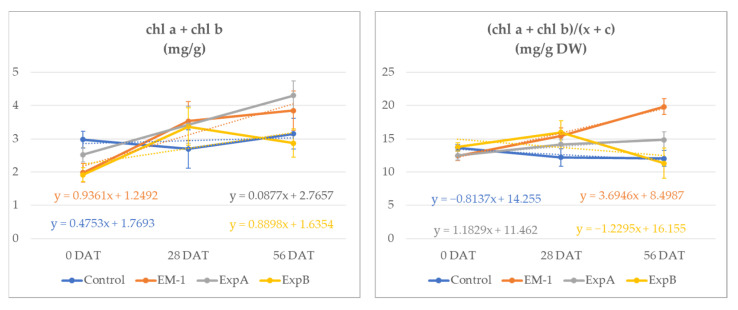
Total chlorophyll content (Chl *a* + Chl *b*) (mg/g DW) and ratio of Chls a and b to total carotenoids (a + b)/(x + c) (mg/g DW) in the leaf material of creeping bentgrass from 0 to 56 DAT for the untreated control, EM-1, ExpA and ExpB.

At 56 DAT, the total chlorophyll content (Chl *a* + Chl *b*) increased in all treatments. However, while the chlorophyll content increased by 5.4% in the untreated control, in the microorganism treatments, the observed increases were more than 55% (+94%, +59%, +67%, respectively) (Figure 1).

The chlorophyll content increases exceeded that of the carotenoids, resulting in significantly higher chlorophyll/carotenoid ratios at 56 DAT in plots treated with EM1 and ExpA (Table 2).

The ratio between the chl *a* and chl *b* content showed a similar effect in the microbiological treatments in comparison with the control. EM1 determined a significant decrease in the Chl a/b ratio of 69% (0–56 DAT). At 56 DAT, the ratio of Chls a and b to total carotenoids (a + b)/(x + c), utilized as the indicator of the greenness of the leaf tissues, indicated higher values for the ExpA and EM-1 treatments. These were both statistically different compared to the control and ExpB, which determined a ratio reduction of 7 and 31%, respectively, from time 0.

Aesthetic values are paramount in evaluating the grass quality of putting greens [17]. An aesthetic assessment was conducted measuring the colour changes of the grassy surface over the end of the experimental trial. At 56 DAT, DGCI values were significantly higher in creeping bentgrass treated with ExpA and ExpB than in the untreated control (Table 3), respectively. Visual differences between the treatments were similarly evident from photographs of the plots (Figure S1).

**Figure 2:**
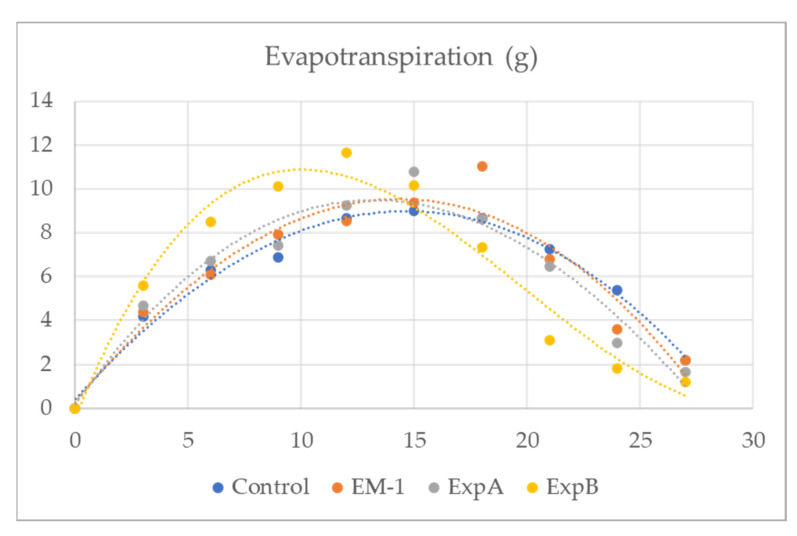
Evapotranspiration (g) of creeping bentgrass from 3 to 30 days after full hydration for grass mats treated with the untreated control, EM-1, ExpA and ExpB.

In addition, plant evapotranspiration in response to biostimulant treatment was investigated in the present study (Figure 2). From 3 to 12 days after full hydration (DAFH), plots treated with ExpB showed a significantly greater evapotranspiration than the untreated control, EM-1 and ExpA, respectively (Table 4). The latter two treatments were not significantly different from the control (Table 4). While the time course of the evapotranspiration of EM-1, ExpA and ExpB appeared to be very similar, it could be observed that the ExpB treatment, on the other hand, had a markedly different behaviour.

**Figure 3:**
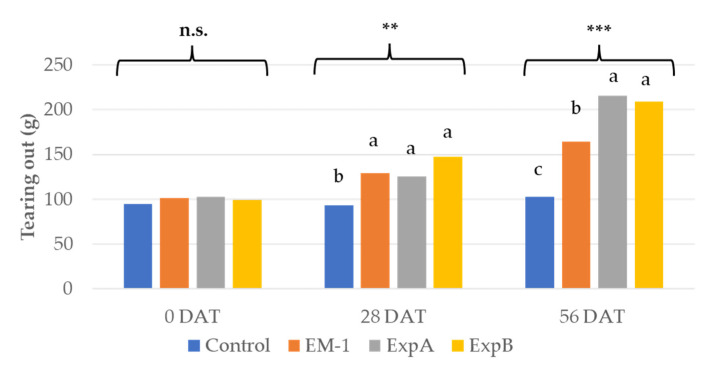
Tearing out resistance at 0, 28 and 56 DAT with the untreated control, EM-1, ExpA and ExpB. Different letters indicate statistically significant different means for *p* < 0.01 (**); *p* < 0.001 (***); n.s., not significant.

Resistance to tearing out is a crucial in turf maintenance [1], and was, thus, tested using the biostimulant treatments. The tearing analysis showed a significant improvement in the mechanical resistance to tearing out at 28 DAT compared to 0 DAT for both ExpA and B (Figure 3). Both of the latter products further improved tearing resistance at 56 DAT compared to 28 DAT. Treatment with EM-1 resulted in an increase in tearing resistance only at 28 DAT (Figure 3).

### 2.2. Biostimulant Effects on the Plant Rhizosphere

Given that thatch build-up is associated with a decreased humus production, organic decomposition and water movement, respectively [2,19], the thickness of the thatch and underlying humus layer were compared both at the start of the experiment and after 56 days. At 0 DAT, no significant differences were observed between the thatch and underlying humus layers of all treatments (Table 5). At 56 DAT, the thickness of the humus layer thickness decreased in the control and increased for EM-1, ExpA, with the highest level of significance reported for Exp B (Table 5). In contrast, the thickness of the thatch layer exhibited an opposite trend. At 56 DAT, the untreated control showed a significantly higher thatch thickness compared to the respective thickness of ExpA and ExpB (21.7, 17.8 and 15.5 mm, respectively) (Table 5).

**Figure 4:**
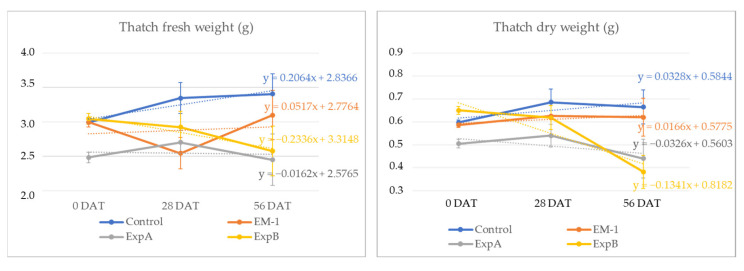
Changes in thatch fresh weight and thatch dry weight from 0 DAT to 56 DAT with the untreated control, EM-1, ExpA and ExpB.

In addition to investigating the thickness of the thatch layer, the thatch fresh weight was examined at 56 DAT and compared with that at 0 DAT for each treatment. In the present experiment, live roots were contained within the thatch material, but the former was shown to constitute a minimal fraction of the overall thatch [17]. Over the two-month period, the thatch fresh weight decreased significantly in response to ExpB. At 56 DAT, the dry mass percentage of the fresh mass was shown to be unchanged for the control, EM-1 and ExpA, but significantly reduced for the ExpB treatment (Figure 4).

**Figure 5:**
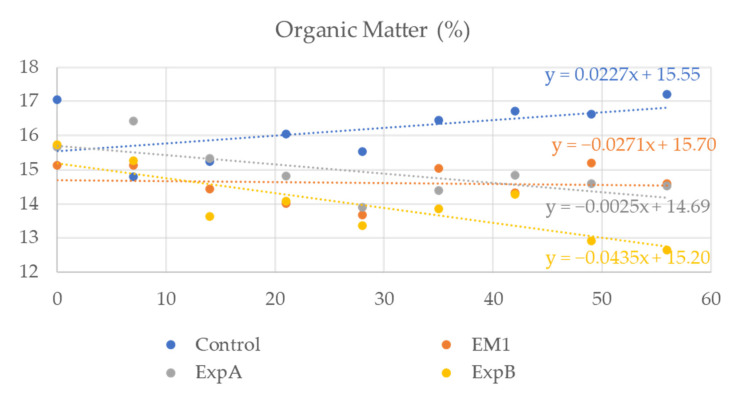
Organic matter content in the soil over the two-month experimental period in response to the untreated control, EM-1, ExpA and ExpB.

To investigate the degradation of the organic component in response to the biostimulant treatment, soil samples spanning both thatch and humus layers were extracted and the organic content determined weekly. At 56 DAT, the organic matter content of the soil was significantly decreased compared to 0 DAT after exposure to ExpB (Figure 5). No significant reduction in organic matter content relative to 0 DAT was evident at 56 DAT for the remaining two biostimulant treatments, but the overall levels were lower than that of the untreated control (Figure 5). In addition, no significant differences in soil carbon and nitrogen were observed.

Arbuscular mycorrhiza fungi (AMF) live in a mutualistic symbiosis with most terrestrial plants, hereby promoting an increased nutrient uptake and plant biomass, as well as resistance to abiotic stress.

**Figure 6:**
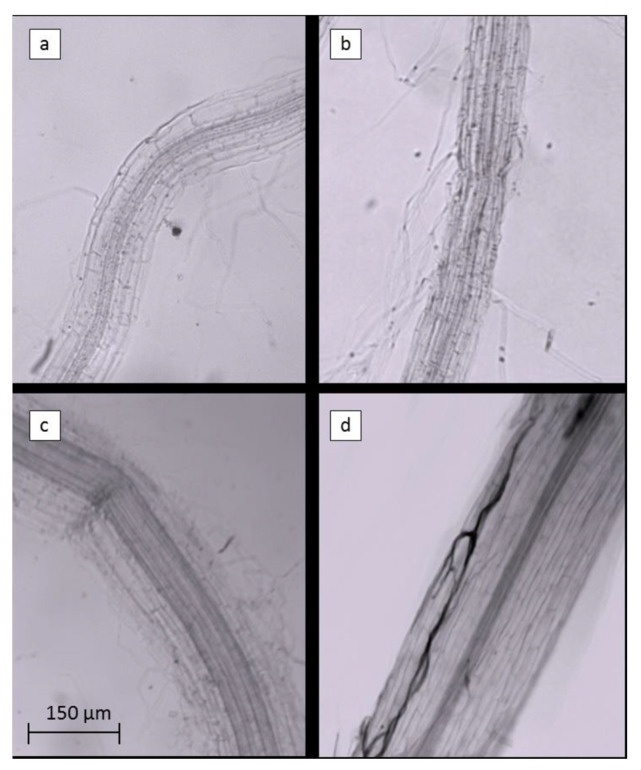
Portions of *Agrostis stoloniferous* L. roots bleached with KOH and treated with Clozaril black E to evidence the Arbuscular Mycorrhizae symbiosis within the root tissues. Roots of control (**a**), EM-1 (**b**), ExpA (**c**) and ExpB (**d**) are shown in order.

The inclusion of AMF in ExpB resulted in a significant increase in the colonization of creeping bentgrass roots (sampled below the thatch layer) at 56 DAT compared to the remaining treatments (Table 6), also illustrated in Figure 6.

## 3. Discussion

Biostimulant research on turf grass is scarce compared to that carried out on agricultural crops [4,8,10,13]. Moreover, there is less published evidence from open-field experiments, and even less pertaining to thatch control-related problems [6,17,18]. The objective of the present study aimed at evaluating the efficacy of a commercial product, EM-1, as well as two biostimulant products (ExpA and ExpB), developed by the present research group, on the widely used temperate turf species *Agrostis stoloniferous* L. Given that field trials provide essential information about biostimulant effects under real-world conditions [29], the experimentation was conducted under open-field conditions, with the aim of improving the characteristics of the plant rhizosphere, whilst simultaneously ensuring improvements to the plant, an aspect considered necessary in turf management. The present results showed that both ExpA and ExpB, identical in microbial composition, were equally effective in improving certain plant characteristics for quality traits in accordance with the new European regulation (EU) 2019/1009 [11]. Significant improvements included increased chlorophyll synthesis and resultant visual quality (DGCI), important for turf aesthetic standards [20], as well as increased resistance to tearing out in comparison to the control after the 56-day experimental period. Of great relevance, the inclusion of humic acids and mycorrhizal fungi to the microbial composition in ExpB significantly improved the plant rhizosphere, which remains an area warranting further investigation [6,17,18].

Improvements to the plant rhizosphere (56 DAT) with ExpB encompassed a significantly decreased thatch layer thickness and fresh mass weight compared to the untreated control. Associated rhizosphere improvements, in accordance with the EU regulation 2019/1009 [11], included a significantly increased humus layer thickness, organic matter degradation and evapotranspiration. Both EM-1 and ExpA afforded benefits that were either marginal or intermediate between that of ExpB and the control. To the best of our knowledge, the present study was the first to report on the effects of a biostimulant product containing both microbial inocula together with humic acids and mycorrhizal fungi for thatch control. Of relevance, it has also been reported that the direct application of ligninolytic enzymes in the form of laccases was promising as a nondisruptive organic approach to managing thatch on turfgrass [18]. After a 6-month application of 2.06 units cm^−2^ of laccase treatment, thatch in a greenhouse pot trial was shown to be reduced by 57.2%. This reduction in thatch was associated with a significant reduction in monosaccharide components derived from structural cellulose and hemicellulose carbohydrates [14]. Interestingly, it was also shown that laccase application for 6 months in a field experiment had residual effects, able to ensure thatch control for a period of one year [15]. This could be achieved through either 12 applications of 0.5 units cm^−2^ laccase (applied once every two weeks for 6 months) or 6 applications 2.0 units cm^−2^ laccase (applied once every four weeks for 6 months) [15]. Although ExpB showed promise for thatch control after 56 DAT, further experiments are required to further investigate the efficacy of this product in terms of duration.

Interestingly, the efficacy after 56 DAT was obtained only with ExpB containing mycorrhizal fungi together with humic acids. Regarding the use of microorganisms alone, previous results showed that lignocellulolytic *Streptomyces* species were ineffective in reducing thatch layers on a golf green over the course of one summer [30]. Similarly, a commercial product containing selected microorganisms with plant extracts was reported to be ineffective in controlling thatch degradation in the short-term [19]. Regarding the use of humic acid-based commercial products alone, under both greenhouse [16] and open field [17] conditions, results showed no decrease in thatch biomass. Although potential for a reduction in thatch thickness was demonstrated, effects were inconsistent over different years. Nonetheless, results of the present investigation clearly showed that the addition of humic acids and mycorrhizal fungi to a consortium of microorganisms rendered ExpB potentially more effective.

It is also important to consider that organic matter accumulation is a crucial problem in modern turfgrass management [18]. In addition, these organic compounds can contribute to the soil water repellency (SWR) that is associated with fungal growth and exudates [31]. Additionally, the SWR can create serious soil water infiltration and potential runoff problems, and contribute to a reduction in turf quality and playability. Aside from meteorological parameters (temperature, solar radiation, humidity and wind velocity) that would have been identical for all treatment plots within the 250 m^2^ experimental area, plant evapotranspiration, facilitating nutrient uptake, is also dependent on both turf properties (thatch leaf weight) and soil-related properties (moisture retention) [26]. It is interesting to note that ExpB reached the maximum peak of evapotranspiration at 12 DAT, earlier than ExpA and the control (which reached this point at 15 DAT) and EM1 (which reached this point at 18 DAT) (Figure 2). This could be very important in reducing possible excessively wet conditions in the turf layer, which can promote the occurrence of grass diseases or stress conditions.

To the best of our knowledge, there have been no reports on the effects of mycorrhizal fungi on thatch control. In the present experiment, the presence of AMF in the root tissue at 56 DAT with ExpB was evident, but no specific effect on thatch control could be inferred. From previous studies, effects of AMF on turfgrass grown under well-watered and water stress conditions showed increased plant biomass and improved morphological traits, respectively [32]. Interestingly, given that water supply is becoming increasingly scarce and costlier, Aalipour et al. [33] reported that the application of mycorrhizal fungi in periods of water scarcity can be considered as one of the most promising methods to improve turfgrass management.

Given the success of AMF’s colonization of creeping bentgrass roots in the present investigation after treatment with ExpB, the benefits of AMF in facilitating the combined beneficial effects of the microbial inoculum and humus in aspects of thatch control may be more evident under water stress conditions. This aspect remains to be investigated and is also warranting of further investigation to substantiate biostimulant claims, for which a minimum of three field trials in the EU are specified under different geoclimatic conditions [29].

The present results showed that despite increases in the thatch layer thickness and fresh biomass weight (and decreased humus layer thickness) in the untreated control plot at 56 DAT, the turf could be considered to be in good condition. The soil pH was maintained throughout the experimental period in the range of 6–7 for all treatments, including the untreated control, a widely-reported optimum pH for microorganism activity and turfgrass growth. Moreover, electrical conductivity, a measure of salinity, was likewise maintained at optimal values in all treatments, suggesting no negative effects on water absorption and nutrient uptake. The soil C/N ratio was also similar in all treatments and close to the value for humus. Hence, the efficacy of ExpB was not attributable to remedying compromised soil conditions, but was a result of the combined activities of the ingredients (microorganisms and humic acids/mycorrhizal fungi) in reducing thatch and organic matter. It would be of interest to test ExpB on the turf grass rhizosphere under compromised conditions, such as excessive thatch build-up and associated problems (more acidic pH, microbial pathogens and localized dry spots). Since the soil pH was maintained around neutral, the presence of microbial pathogens was not assessed in the present study. Although microbial inocula are reported to be effective in controlling soil pathogens [23], this aspect remains to be investigated using both ExpA and ExpB.

The results of the present study also showed improvements in ExpB to plant characteristics. Only ExpB, distinguishable for the addition of humic acid and mycorrhizal fungi, was shown to increase leaf biomass. Previous results demonstrated an increase in plant biomass under well-watered conditions, attributable to the presence of the mycorrhizal fungi [32]. Although humic acid products have been shown to improve shoot and, more specifically, root biomass [26,27], rooting properties were not examined in the present study. Relating to additional improvements to the plant characteristics, ExpA and ExpB were equally effective at improving the visual quality, attributable to the increased chlorophyll content. Regarding the improved visual quality and/or chlorophyll, previous reports on turf grasses have all unanimously shown an improved visual quality after exposure to the following: microbial treatments alone [13,23,25], humic acids alone [4,26,27,32], combined treatments with beneficial microorganisms and humic acids [23], combined treatments with beneficial microorganisms, humic acids and AMF [20], AMF alone [32], plant growth regulators [34], plant extracts [23,34] and amino acids [9,13], respectively. Collectively, aspects pertaining to the visual qualities appeared to respond more readily to a wider selection of biostimulants. The results of the present study also demonstrated an equivalent, significantly improved resistance to tearing out at 56 DAT with both ExpA and ExpB, relative compared to the control and EM-1. However, EM-1 also showed significantly improved resistance to tearing out compared to the control, corroborating previous findings on ryegrass [1]. Increased resistance to tearing out is particularly favourable for sports turfs, and the colonization of the roots by various *Lactobacillus* species was also reported, showing that microbial inoculum enhanced the morphophysiological effects on plant shoots and roots [1].

## 4. Materials and Methods

### 4.1. Biostimulant Preparations

An untreated control and three biostimulant treatments (one commercial and two experimental products) were investigated, respectively. The untreated control was composed of distilled water. The commercial product used was EM-1 (EM Research Organization (EM-RO^®^), Okinawa, Japan), a bacterial solution containing a mixture of lactic acid bacteria, yeasts and photosynthetic bacteria (https://www.emrojapan.com/products/accessed on 18 December 2022). The “activation” of the product EM-1^®^ was performed according to the manufacturer’s instructions. The product was suspended in distilled water (5% *v*/*v*), together with 5% (*v*/*v*) of molasses, and maintained in an incubator at 35 °C for 5 days. Before the application, the product was further diluted in water to attain a final dilution of 1:500 [1]. The two experimental treatments were formulated by the Department of Agricultural and Food Sciences, University of Bologna, and labelled as ExpA and ExpB, respectively.

Both ExpA and ExpB contained the same microbial consortium (containing selected bacterial and fungal strains) that were cultured individually according to their respective requirements, and were then mixed prior to the experimental inoculation. Specific organisms, levels and viability were unavailable due to product confidentiality. Mycorrhizal fungal spores and humic acid were also added to ExpB, which was patented (Patent Cooperation Treaty, 2017) [35] under the International Publication Number WO 2017/017633 A1. Similar to EM-1, ExpA and ExpB were, respectively, diluted in water to attain a final dilution of 1:500.

### 4.2. Experimental Field Design, Biostimulant Applications and Maintenance Activities

The field trial was conducted on the putting green of the Modena Golf & Country Club (Modena, Italy, 44.5484° N, 10.9039° E). The experimental field (250 m^2^) was located at the end of hole number six. The putting green was built and managed according to USGA guidelines [36], using artificially constructed soil, composed of a 80% sand and 20% peat mixture layer (30 cm) over a gravel layer (10 cm). At the beginning of the field trial, the putting green soil showed a standard value of ECe (1.73 ± 0.27 mS cm^−1^) and a neutral—slightly alkaline value of pH (7.2 ± 0.7). The putting green consisted entirely of the herbaceous creeping bentgrass (*Agrostis stolonifera* var. Penncross).

The field trial was carried out according to a randomized complete block design with three replicate plots per treatment, respectively, each with dimensions of 2 m^2^ (Figure S2). A distance of 2 m separated each experimental plot in order to lower the risk of contamination between the different treatments. The biostimulant treatments were applied as solutions from a shoulder-pump sprayer calibrated to deliver 0.5 L m^−2^. The application of the treatments to each plot was performed at the closing time of the golf club to avoid possible dispersion induced by trampling athletes. Directly after the application of the respective treatments, the irrigation system in the experimental area was activated for 2 min to facilitate the movement of the treatments from the leaves into the deeper layers of the turf. The water control was applied according to the same protocol as the biostimulant treatments. All treatments were performed weekly for a period of two months from the starting date on 10 July 2012.

Temperature and precipitation data during the experimental trial were provided by the weather station in Maranello, near the golf course (Modena, Italy) and are reported in Figure S3. Maintenance activities, including the aeration of the lawns, as well as fungicide and pesticide applications were suspended for the 2-month duration of the experiment. Mowing activities were reduced from five times a week to once a week. Irrigation remained unchanged. A starter fertilizer product with an NPK of 16-25-12 (Lebanon ProScape, Herbatech, Italy) was applied at a dosage of 20 g m^−2^ at the end of March before the initiation of the experimental trial.

### 4.3. Biostimulant Effects on the Plant

#### 4.3.1. Leaf Biomass

Leaves were cut and weighed to obtain the fresh biomass. The leaf material collected on a surface area of 2.5 cm × 10 cm was dried in an oven at 50 °C for 4 days and the dry biomass was determined.

#### 4.3.2. Leaf Pigment Analyses

At 0 and 56 DAT, the photosynthetic pigments, chlorophylls a and b, as well as total carotenoids (xanthophylls and carotenes (x + c)), were extracted with 100% acetone and determined spectrophotometrically (Shimadzu UV–VIS scanning spectrophotometer UV-2001 PC, Kyoto, Japan) according to Lichtenthaler et al. [37]. Calculations of leaf pigments were described by Lichtenthaler et al. [37] as follows:

- Chlorophyll a (μg/mL) = 11.24 × A661.6 − 2.04 × A644.8;
- Chlorophyll b (μg/mL) = 20.13 × A644.8 − 4.19 × A661.6;
- Carotenoids (x + c) (μg/mL) = (1000 × A470 − 1.90 × Chl a − 63.14 × Chl b)/214.

#### 4.3.3. Turfgrass Colour Analysis

The colour of the grass surface was measured at the onset (0 DAT) and at the end of the experiment (56 DAT), respectively, by capturing 2000 × 2000-pixel images using a Canon EOS 350D reflex camera. The capture homogeneity of the photographs was ensured by maintaining the same diaphragm aperture settings as well as exposure times (shutter speed = 1/800 s; diaphragm aperture F = 16; sensitivity = ISO 1600; focal length = 18 mm). Moreover, differences in light, attributable to meteorological conditions, were reduced by normalizing the images with a professional photographic program (Adobe Photoshop Lightroom 4). The average red, green and blue levels (RGB) of the digital images were calculated using the software APS ASSESS 2.0 and then converted to hue, saturation and brightness (HSB) values. A dark green colour index (DGCI) was created from the HSB values to obtain a single representative value of the relative dark green colour of the image [38]. The index was created using the following equation:

DGCI value = [(H − 60)/60 + (1 − S) + (1 − B)]/3.

The DCGI value ranged from zero to one with higher values corresponding to a darker green colour.

#### 4.3.4. Evapotranspiration

Modifying and adapting the microlysimeter methods described by Kool et al. [39], evapotranspiration analyses were carried out on grass mat samples extracted over a surface area of 2.5 cm^2^ and a depth of 5 cm within each plot. At 56 DAT, grass mat samples were weighed and then watered (avoiding leaves and stolons) until the soil water content reached 100% of field capacity. The mat was then sealed with parafilm, leaving only the upper grass surface free, to ensure that water loss was due only to evapotranspiration. The grass mat samples were placed in a growth chamber with artificial lighting with a day/night photoperiod (16/8 h) and temperature of 28/20 °C. The weight loss of each grass mat, attributable to the evapotranspiration, was measured every three days at 17:00 until the soil was completely dried.

#### 4.3.5. Tearing

Shoot resistance to tearing out was measured using a dynamometer (PCE Instrument, Lucca, Italy) according to the manufacturer’s instructions. The method employed consisted of measuring the force required to tear the grass shoot from the soil. The end of the dynamometer, equipped with a pair of tweezers, was attached to the base of the stem of each plant sampled and three replicate measurements were obtained for each treatment at 0, 28 and 56 DAT, respectively.

### 4.4. Plant Rhizosphere Analyses

#### 4.4.1. The Soil Profile

The thatch and underlying humus layer were, respectively, measured weekly for the entire duration of the experimental period (56 days). An extractor tool was used to expose a segment of the underlying soil profile (width 2.5 cm × length 10 cm × depth 10 cm). The measuring point was set at 5 mm below the leaf surface, extending downwards to include the height measurements of both the thatch and humus layers, respectively. The average height of both the thatch and humus layers was calculated as a mean value of 10 individual points along the horizontal section of the respective layers (Figure S4) at 0 and 56 days after the treatments (DAT).

#### 4.4.2. Thatch Biomass

Thatch (intermingled organic layer of dead and living roots) mixed with soil is typically referred to as the “mat”. Mat samples were collected by cutting away leaves and stolons at 0.5 mm from the surface. Mat samples were then washed using a lab sieve (Retsch^®^, sieve mesh 0.5 mm) to remove the soil component. Thereafter, thatch (including living roots) was collected to measure the fresh weight. Samples were then dried for 5 days at 50 °C to determine the dry biomass. Thatch biomass measurements were conducted at 0 and 56 DAT.

#### 4.4.3. Arbuscular Mycorrhiza Fungi Analysis

At 56 DAT, roots were collected from 5 cm diameter soil cores and the presence of *Arbuscular mycorrhiza* fungi (AMF) within the root tissues was analysed according to Brundrett et al. [40]. Roots were rinsed thoroughly with distilled water, cut into 1 cm long segments, added to a 10% (*w*/*v*) KOH solution in autoclave-resistant jars and autoclaved for 15 min at 121 °C. Root samples were then rinsed with several changes of deionized water and transferred into a staining solution composed of 80% lactic acid, glycerine and distilled water (1:1:1) with 0.1% (*w*/*v*) Clozaril black E (Sigma Aldrich, Milan, Italy). For each sample, 200 randomly selected root segments were used. The roots (50 segments at a time) were spread out evenly in a Petri dish (90 mm diameter, Sterlin^®^) with grid lines marked on the bottom of the dish (1 cm x 1 cm squares). Under a dark field phase contrast microscope (MEIJI, Chuo City, Japan, 400 × magnification), vertical and horizontal gridlines were scanned and the presence or absence of AMF was recorded at each point where the roots intersected a line. The root segments were respread and examined four times. Finally, the colonization was calculated as the number of intersections with AMF divided by the total number of root–grid intersections examined, as described previously [40].

### 4.5. Statistical Analysis

The analysis of variance (ANOVA) and comparisons of means (using Tukey’s HSD tests at *p* < 0.05) were performed using the statistical software CoSTAT Version 6.002 (CoHort Software, Monterey, CA, USA).

## 5. Conclusions

ExpB, containing a combination of both microbial inoculum with humic acid and mycorrhizal fungi, collectively improved both rhizosphere and plant characteristics. Under the same meteorological conditions (water and temperature), ExpB significantly decreased the thatch layer thickness and fresh mass weight, and increased the humus layer thickness, organic matter decomposition, evapotranspiration, leaf dry weight, visual quality, chlorophyll content and resistance to tearing out, respectively. ExpA (microbial inoculum only) was only as effective as ExpB in improving the visual quality, chlorophyll content and resistance to tearing out. Hence, ExpB showed promising potential as a biostimulant towards relieving the negative environmental impacts and high costs associated with turf maintenance-related activities. Additional experimentation is required under less well-maintained turfgrass conditions or abiotic stress to further study the effect of ExpA and ExpB on the rooting properties and verify the efficacy of the products.

## 6. Patents

ExpA and ExpB are described in UNIBO Patent WO 2017/017633 Al.

## Figures and Tables

**Table 1:** Changes in fresh and dry leaf weight, respectively, at 0, 28 and 56 DAT with the untreated control, EM-1, ExpA and ExpB.

	Treatments	Fresh Leaf Weight (g)	Dry Leaf Weight (g)
0 DAT		ns	ns
	Control	812.7	207.2
	EM-1	767.5	218.9
	ExpA	810.4	217.4
	ExpB	751.8	227.3
28 DAT		ns	ns
	Control	850.5	236.5
	EM-1	814.9	260.0
	ExpA	835.6	287.6
	ExpB	867.1	257.4
56 DAT		**	*
	Control	821.9 (a)	222.8 (b)
	EM-1	630.5 (b)	209.8 (b)
	ExpA	836.2 (a)	252.1 (ab)
	ExpB	947.8 (a)	305.1 (a)

Different letters indicate statistically significant different means for *p* < 0.05 (*); *p* < 0.01 (**); ns, not significant.

**Table 2:** Comparison of total chlorophyll (chl *a* + chl *b*), the ratio of chl *a* and chl *b* and carotenoids (carotene and xanthophylls: c + x) in the leaf material of creeping bentgrass at 0, 28 and 56 DAT for the untreated control, EM-1, ExpA and ExpB.

	Treatments	chl *a* + chl *b* (mg/g DW)	chl *a*/chl *b* (mg/g DW)	(chl *a* + chl *b*)/c + x (mg/g DW)
0 DAT		**	ns	ns
	Control	2.98 (a)	2.01	13.65
	EM-1	1.98 (b)	2.13	12.42
	ExpA	2.52 (ab)	2.06	12.48
	ExpB	1.92 (b)	1.89	13.79
28 DAT		ns	ns	ns
	Control	2.69	1.96	12.20
	EM-1	3.53	1.81	15.43
	ExpA	3.42	1.81	14.16
	ExpB	3.37	1.92	15.98
56 DAT		*	*	*
	Control	3.15 (ab)	1.53 (bc)	12.03 (b)
	EM-1	3.85 (ab)	1.47 (c)	19.81 (a)
	ExpA	4.30 (a)	1.81 (ab)	14.85 (ab)
	ExpB	2.87 (b)	1.84 (a)	11.33 (b)

Different letters indicate statistically significant different means for *p* < 0.05 (*); *p* < 0.01 (**); ns, not significant.

**Table 3:** Dark green colour index (DGCI) of creeping bentgrass at 56 DAT in response to exposure to the untreated control, EM-1, ExpA and ExpB.

	Treatment	DGCI
		*
56 DAT	Control	0.36 (b)
	EM-1	0.47 (ab)
	ExpA	0.55 (a)
	ExpB	0.55 (a)

Different letters indicate statistically significant different means for *p* < 0.05 (*).

**Table 4:** Evapotranspiration (g) of creeping bentgrass from 3 to 30 days after full hydration (DAFH) for grass mats treated with the untreated control, EM-1, ExpA and ExpB.

	Treatments				
DAFH	Control	EM-1	ExpA	ExpB	Significance
3	4.2 (b)	4.4 (b)	4.7 (b)	5.6 (a)	**
6	10.5 (b)	10.5 (b)	11.4 (b)	14.1 (a)	*
9	17.3 (b)	18.4 (ab)	18.8 (ab)	24.2 (a)	*
12	26.0 (b)	27.0 (b)	28.1 (b)	35.8 (a)	*
15	35.0	36.3	38.8	46.0	ns
18	43.7	47.4	47.5	53.3	ns
21	50.9	54.1	54.0	56.5	ns
24	56.3	57.8	56.9	58.3	ns
27	58.5	59.9	58.6	59.5	ns
30	62.6	61.3	61.6	62.5	ns

Different letters indicate statistically significant different means for *p* < 0.05 (*); *p* < 0.01 (**); ns, not significant.

**Table 5:** Changes in thatch thickness and thatch fresh and dry weight, respectively, at 0, 28 and 56 DAT with the untreated control, EM-1, ExpA and ExpB.

	Treatments	Humus Thickness (mm)	Thatch Thickness (mm)	Thatch Fresh Weight (g)	Thatch Dry Weight (g)
0 DAT		ns	ns	***	ns
	Control	18.4	18.30	3.00 (a)	0.60
	EM-1	18.1	20.70	3.00 (a)	0.59
	ExpA	17.3	18.50	2.48 (b)	0.51
	ExpB	18.3	21.10	3.04 (a)	0.65
28 DAT		ns	ns	*	ns
	Control	17.9	19.70	3.35 (a)	0.69
	EM-1	18.9	21.10	2.54 (b)	0.63
	ExpA	18.0	19.30	2.70 (b)	0.54
	ExpB	20.5	19.90	2.92 (ab)	0.62
56 DAT		**	*	*	*
	Control	16.7 (c)	21.70 (a)	3.41 (a)	0.67 (a)
	EM-1	22.0 (ab)	19.40 (ab)	3.10 (ab)	0.62 (ab)
	ExpA	19.0 (bc)	17.80 (ab)	2.45 (b)	0.44 (bc)
	ExpB	24.8 (a)	15.50 (b)	2.58 (b)	0.38 (c)

Different letters indicate statistically significant different means for *p* < 0.05 (*); *p* < 0.01 (**); *p* < 0.001 (***); n.s., not significant.

**Table 6:** Arbuscular mycorrhiza fungi (AMF) colonization of creeping bentgrass roots at 56 DAT in response to exposure to the untreated control, EM-1, ExpA and ExpB.

Treatment	AMF % in Roots
Control	5.5 ± 1.6
EM-1	6.2 ± 1.4
ExpA	4.3 ± 3.7
ExpB	28.7 ± 5.1

## Data Availability

Not applicable.

## Supplementary Materials

The following supporting information can be downloaded at: https://www.mdpi.com/article/10.3390/plants12030539/s1, Figure S1: visual differences between the treatments at 56 DAT from photographs of the plots; Figure S2: randomized complete block design with three replicate plots per treatment; Figure S3: temperature and precipitation data recorded during the experimental trial and provided by the weather station in Maranello, near the golf course (Modena, Italy); Figure S4: height of both the thatch and humus layers calculated as a mean value of 10 individual points along the horizontal section of the respective layers at 0 and 56 days after the treatments (DAT).
