# Supplementary material for: Biostimulants for Sustainable Management of Sport Turfgrass
Source: Plants (Basel). 2023 Jan 24;12(3):539. doi: 10.3390/plants12030539 (PMC9921520; doi:10.3390/plants12030539)

MODENA GOLF & COUNTRY CLUB

FIELD TRIAL in *Agrostis stolonifera* putting green

Suspension of all managements scheduled (mechanicals and chemicals)

-30 days after treatments

0 days after treatments

28 days after treatments

56 days after treatments

Treatments tested during the field trials:

**CONTROL Without treatment**	**EM1 commercial product**	**ExpA**	**ExpB**

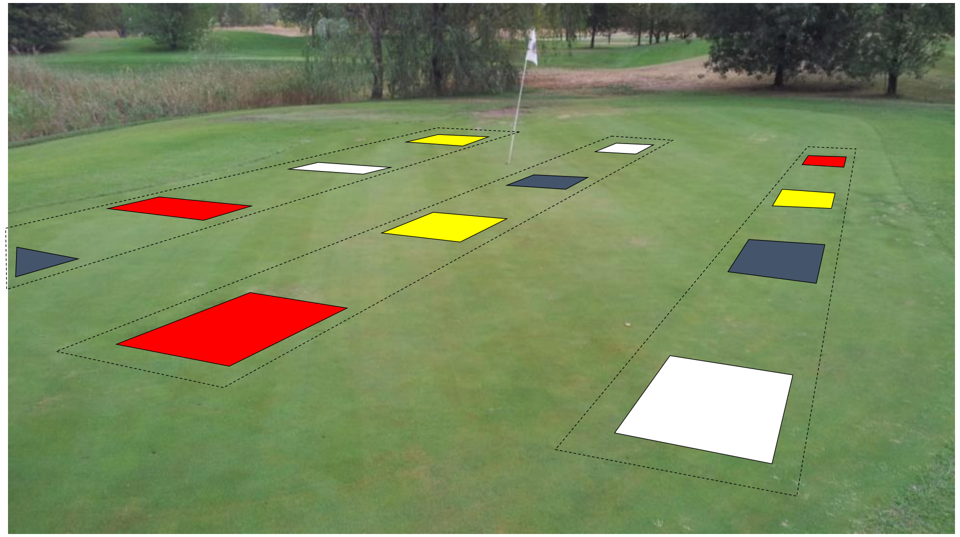

Supplement: Supplementary file 1 [file plants-12-00539-s001.zip › Supplementary_ Figure S2.pdf]
