# Supplementary figures and images for: Biostimulants for Sustainable Management of Sport Turfgrass
Source: Plants (Basel). 2023 Jan 24;12(3):539. doi: 10.3390/plants12030539 (PMC9921520; doi:10.3390/plants12030539)

0

7

14

21

28

35

42

49

56

DAT

CONTROL

EM-1

ExpA

ExpB

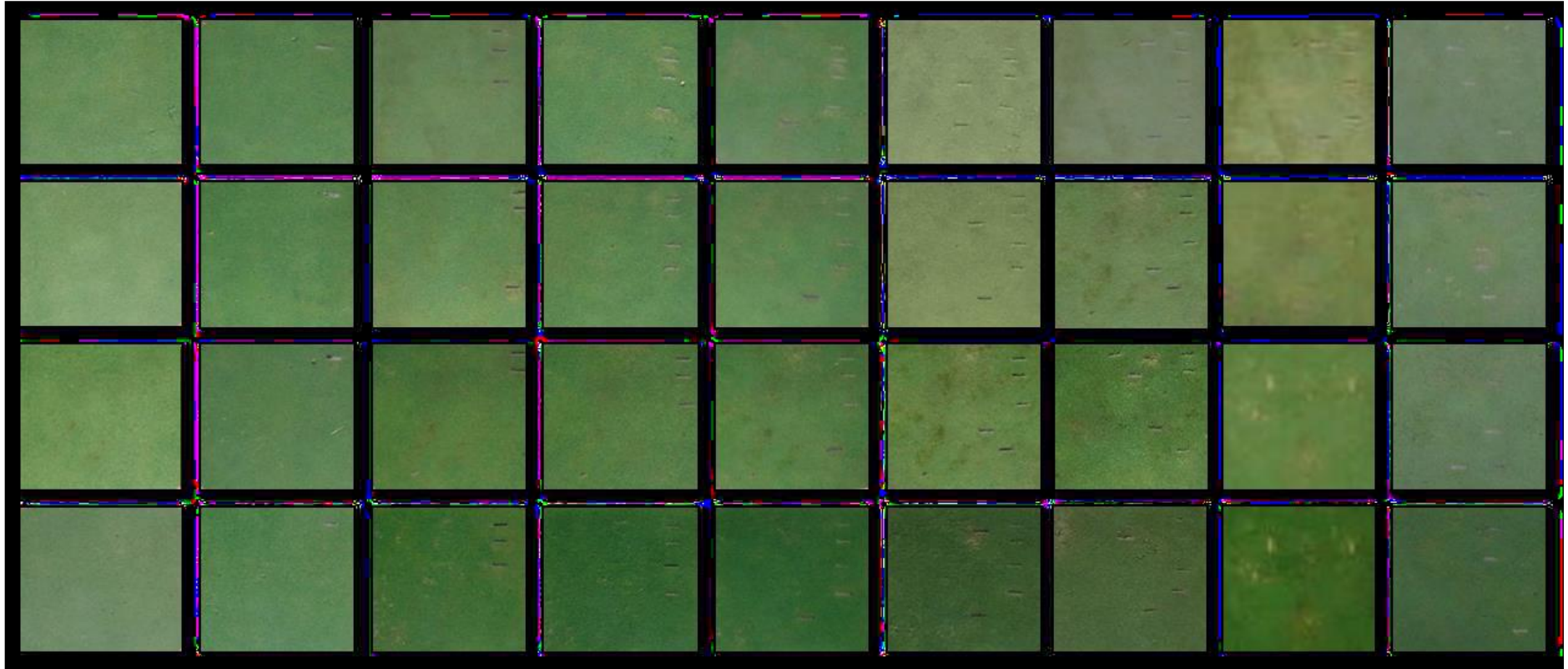

Supplement: Supplementary file 1 [file plants-12-00539-s001.zip › Supplementary_ Figure S1.pdf]

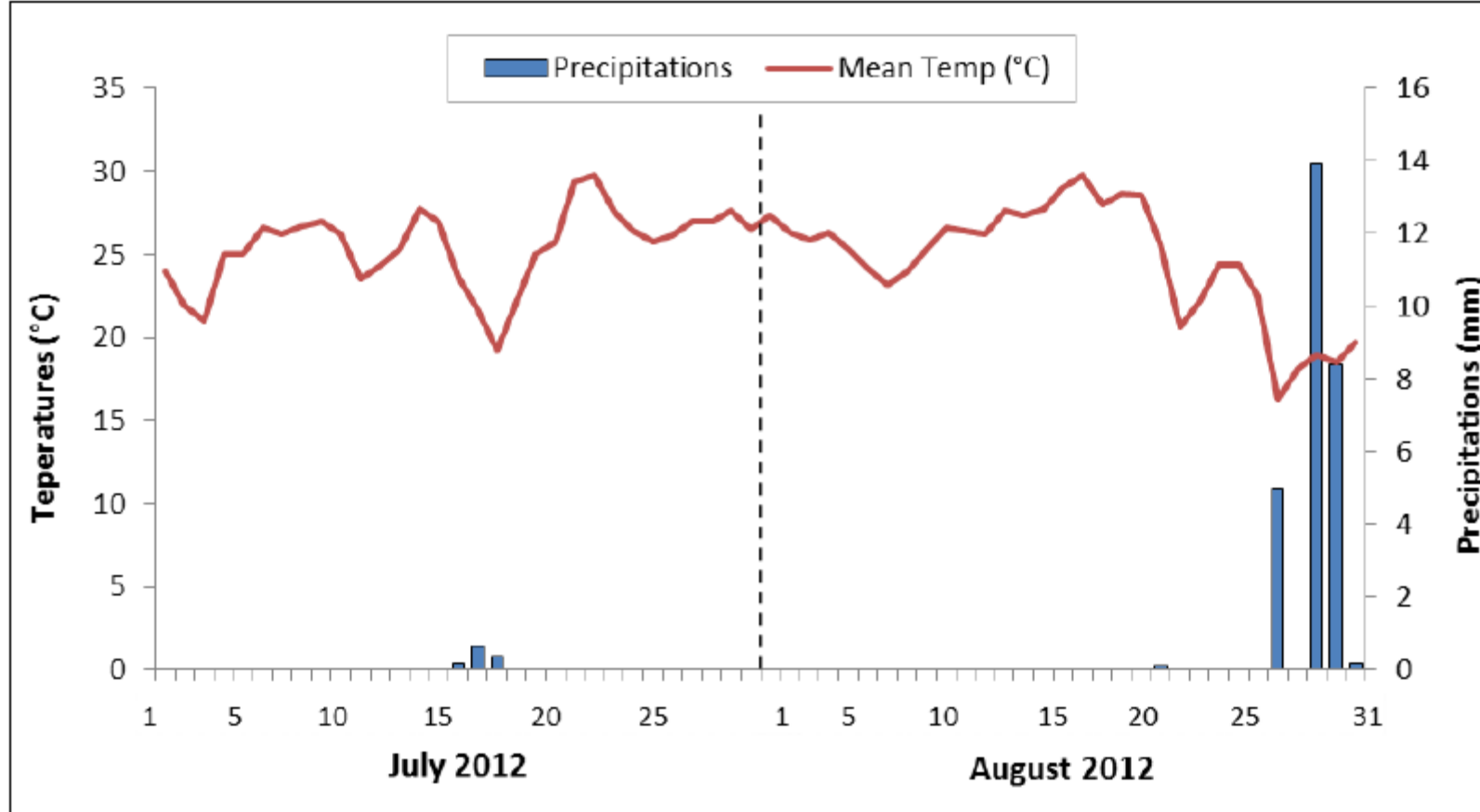

Supplement: Supplementary file 1 [file plants-12-00539-s001.zip › Supplementary_ Figure S3.pdf]

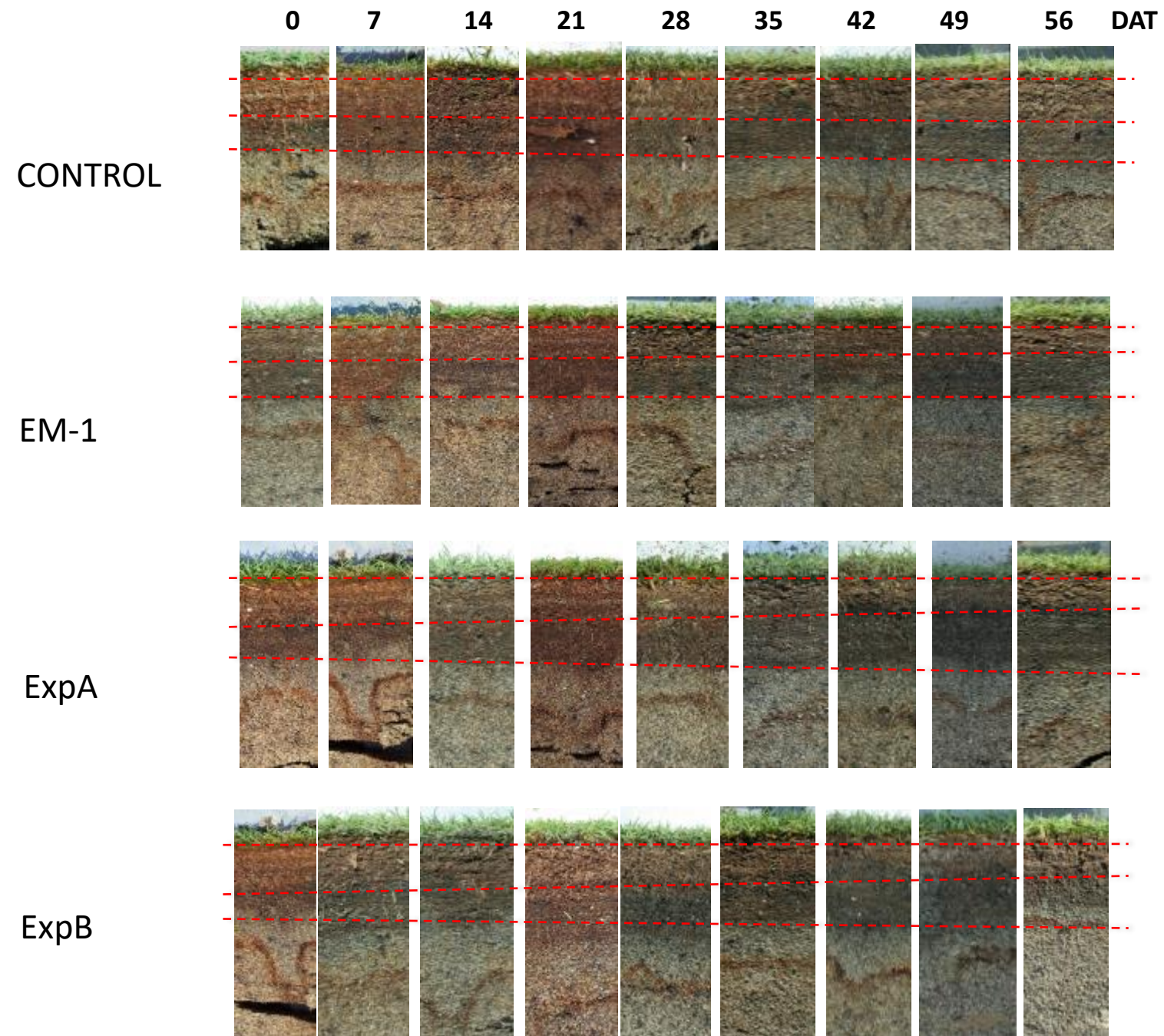

Supplement: Supplementary file 1 [file plants-12-00539-s001.zip › Supplementary_ Figure S4.pdf]
